# SARS-CoV-2 is rapidly inactivated at high temperature

**DOI:** 10.1007/s10311-021-01187-x

**Published:** 2021-02-03

**Authors:** Jennifer Biryukov, Jeremy A. Boydston, Rebecca A. Dunning, John J. Yeager, Stewart Wood, Allison Ferris, David Miller, Wade Weaver, Nathalie E. Zeitouni, Denise Freeburger, Paul Dabisch, Victoria Wahl, Michael C. Hevey, Louis A. Altamura

**Affiliations:** National Biodefense Analysis and Countermeasures Center (NBACC), Operated By Battelle National Biodefense Institute (BNBI) for the U.S. Department of Homeland Security Science and Technology Directorate, Fort Detrick, MD 21702 USA

**Keywords:** SARS-CoV-2, Coronavirus, COVID-19, Environmental decay, Heat, Viral inactivation

## Abstract

In the absence of a vaccine, preventing the spread of the severe acute respiratory syndrome coronavirus 2 (SARS-CoV-2) is the primary means to reduce the impact of the 2019 coronavirus disease (COVID-19). Multiple studies have reported the presence of SARS-CoV-2 genetic material on surfaces suggesting that fomite transmission of SARS-CoV-2 is feasible. High temperature inactivation of virus has been previously suggested, but not shown. In the present study, we investigated the environmental stability of SARS-CoV-2 in a clinically relevant matrix dried onto stainless steel at a high temperature. The results show that at 54.5 °C, the virus half-life was 10.8 ± 3.0 min and the time for a 90% decrease in infectivity was 35.4 ± 9.0 min. These findings suggest that in instances where the environment can reach temperatures of at least 54.5 °C, such as in vehicle interior cabins when parked in warmer ambient air, that the potential for exposure to infectious virus on surfaces could be decreased substantially in under an hour.

## Introduction

In the absence of a therapeutic or vaccine, preventing person-to-person spread of SARS-CoV-2 is the primary means to reduce the impact of COVID-19. While the relative contribution of potential routes of exposure remains unclear, multiple studies have reported the presence of SARS-CoV-2 genetic material on surfaces suggesting that fomite transmission is possible (Ben-Shmuel et al. 2020; Chia et al. 2020; Guo et al. 2020; Ong et al. 2020; Santarpia et al. 2020). Experimental studies to evaluate the influence of environmental factors on SARS-CoV-2 stability in the air and on surfaces are critical to understanding the risk of transmission under different conditions (Sharma et al. 2020). Previously, we evaluated the stability of SARS-CoV-2 on three nonporous surfaces in two suspension matrices and under different environmental conditions (Biryukov et al. 2020; Ratnesar-Shumate et al. 2020). These results demonstrated that increasing temperature and/or humidity increased the decay rate of SARS-CoV-2, and that virus decay could be fit to a relatively simple regression model that was valid within a range of experimentally tested conditions. In these previous studies, we evaluated temperatures up to 35 °C, but even higher temperatures may occur outdoors or in unconditioned environments, which can result in accelerated decay. For example, parked vehicles in sunlight can undergo a greenhouse effect, trapping long-wave thermal radiation within the vehicle, resulting in rapid and significant elevations in cabin temperatures. In one study, cabin temperatures increased by an average 22 °C after one hour when outside air temperatures ranged from 22 to 36 °C, resulting in approximate cabin temperatures of 44 to 58 °C (McLaren et al. 2005). In another study, a passenger car left in a parking lot in sunlight for up to 90 min resulted in cabin temperatures of 51 to 56 °C (Wang et al. 2020). Taking a more active approach to heating vehicles, the Ford Motor Company released a news brief describing a software solution for Police Interceptor Utility vehicles (utilized by law enforcement agencies throughout the United States) that would increase the interior temperature above 56.1 °C (133 *°*F) for 15 min (Ford Motor Company 2020). Ford reported that heat treatment of the vehicle’s interior for 15 min reduced the concentration of infectious coronaviruses by approximately 99%, although the methodology and data from those studies have not been made publicly available. Here, we have expanded upon our previous work and evaluated the stability of SARS-CoV-2 at a higher temperature relevant to vehicle interiors that are parked outside in direct sunlight, as these data may be useful to inform approaches for sanitizing vehicle interiors, including automobiles, buses, and commuter trains.

## Materials and methods

*Cells* Vero (ATCC® CCL-81TM) cells were cultured at 37 °C and 5% CO_2_ in complete growth medium (gMEM), as described previously (Biryukov et al. 2020). Vero cells were used to propagate SARS-CoV-2 and in virus microtitration assays. Cells were seeded into 96-well clear bottom plates for virus microtitration assays using a VIAFILL reagent dispenser (INTEGRA Biosciences Corp.).

*Virus* The following reagent was deposited by the Centers for Disease Control and Prevention and obtained through BEI Resources, NIAID, NIH: SARS-related coronavirus 2, isolate USA-WA1/2020, NR-5228. The virus was obtained as passage four material and passaged once in Vero cells to generate a passage five master stock. Virus master stock was used to generate passage six working stock that was used for this work. This material was prepared as previously described by clarification of freeze-thawed infected Vero cell monolayers and culture supernatants (Biryukov et al. 2020). Virus was aliquotted and stored at − 80 °C prior to use in experiments.

*Virus Microtitration Assay* Virus containing samples were serially diluted (10^–1^ through 10^–4^) in 96-well clear bottom plates containing confluent monolayers of Vero cells. For each sample, a total of ten replicate wells were infected and titrated. Infected plates were incubated for four days at 37 °C and 5% CO_2_ followed by microscopic visualization of virus-induced cytopathic effects (CPE) at each dilution, relative to a negative, media only, control. The median tissue culture infectious dose (TCID_50_) for each sample was estimated using the Spearman-Karber method (Spearman 1908; Karber 1931).

*Test matrix, surface decay test system, and design of experiments* SARS-CoV-2 were diluted 1:10 in simulated saliva, as described previously (Biryukov et al. 2020), to approximate the behavior of virus in a relevant bodily fluid. The biological decay rate of virus at specific environmental conditions was completed as previously described (Biryukov et al. 2020). Briefly, virus-laden wet droplets (5 µL) were deposited onto 304 stainless steel coupons and either immediately incubated at 54.5 °C and 20% relative humidity (RH) or allowed to dry followed by incubation at 54.5 °C and 20% RH. Coupons were incubated at condition for up to 2 h. The initial virus titers were determined by placing virus-laden coupons (containing either wet or dry droplets) into 4 mL gMEM, vortexing for 30 s at 2400 rpm and then quantifying infectivity by virus microtitration assay. Three coupons were retrieved at various time points following incubation and infectious virus extracted from the coupon and quantified as described above.

*Statistical analysis* A logarithm (base 10) transformation was applied to the TCID_50_ data, and a linear model was fit using JMP’s general linearized model with an identity link function and normal distribution. Only results where at least one well scored positive for virus-induced CPE were included in the model. The half-life (*t*_1/2_) was determined using Eq. 1 where *k* is the log_10_ TCID_50_/mL loss per hour. The time for 90% loss was equal to the reciprocal of the slope of the log_10_ linear regression.

1 $$t_{1/2} = \frac{{\log_{10} 2}}{k}$$

A Student’s *t*-test was used to determine that there was no difference in mean half-life between wet and dry droplets, and therefore, these trials were combined to determine the average half-life of virus at 54.5 °C and 20% RH.

## Results and discussion

**Fig. 1:**
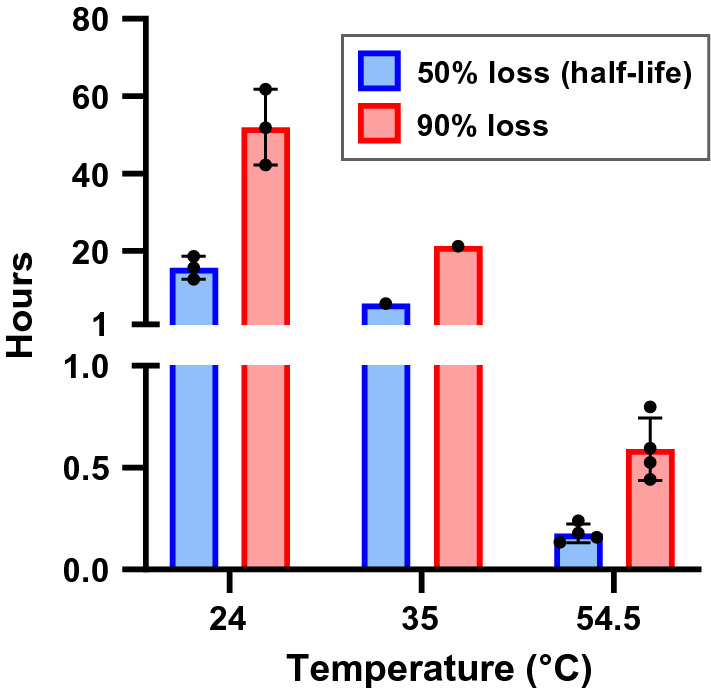
Impact of temperature on the surface decay of the the severe acute respiratory syndrome coronavirus 2 (SARS-CoV-2)

To evaluate the stability of SARS-CoV-2 at a high temperature achievable within interiors of vehicles parked in direct sunlight, we employed the same methodology and environmental chamber as used in our study demonstrating the effects of temperature and relative humidity (RH) on virus stability (Biryukov et al. 2020). SARS-CoV-2 was diluted 1:10 in artificial saliva to simulate a clinical matrix relevant to COVID-19 transmission. In one set of tests, 5 µL droplets of viral suspension were deposited onto stainless steel coupons and immediately incubated at 54.5 ± 0.2 °C and 20 ± 0.4% RH for up to 2 h. In a second set of tests, 5 µL droplets were deposited onto stainless steel coupons and allowed to dry at ambient conditions (23.5 ± 0.03 °C and 47.2 ± 0.2% RH) prior to incubation at 54.5 ± 0.2 °C and 20 ± 0.4% RH for up to 2 h. For each test, virus was recovered from three replicate coupons into culture media at predetermined time points and the concentration of infectious virus remaining was quantified using a cell-based microtitration assay. Linear regression analysis, plotting log_10_-transformed viral infectivity data over time, was used to calculate the decay rate (*k*), half-life (*t*_1/2_), and the time for a 90% decrease in viral infectivity to occur (Table 1 and Fig. 1). Virus suspended in saliva had similar half-lives (± SD) at 54.5 °C and 20% RH for both the virus that was allowed to dry at ambient conditions and wet virus placed immediately at high temperature (dry *t*_1/2_ = 12.6 ± 2.6 min; wet *t*_1/2_ = 8.8 ± 1.1 min; *P* = 0.19 when compared using an unpaired *t*-test and assuming equal variances). For comparison, we previously reported that decay on stainless steel at lower temperatures took significantly longer, with mean half-lives of wet-deposited virus at 24 °C or 35 °C and 20% RH of 15.6 ± 3.0 and 6.4 h, respectively (Biryukov et al. 2020).

**Table 1:** Surface stability of the severe acute respiratory syndrome coronavirus 2 (SARS-CoV-2) at 20% relative humidity (RH)

T (°C)	RH (%)	No. tests	*k*
24*	20	3	0.02 ± 0.004
35*	20	1	0.05
54.5	20	4	1.7 ± 0.42

*T*, temperature; *RH*, relative humidity; *k*, decay rate (log_10_ TCID_50_/mL loss per hour)

For each test, three replicate coupons were evaluated at each time point, and the data were fit to a linear model

*Data were previously published, and are included here for comparison

SARS-CoV-2 was diluted 1:10 in simulated saliva and then 5 µL droplets were deposited onto stainless steel coupons, which were incubated within an environmentally controlled chamber at 20% RH and at various temperatures. During each experiment, three test coupons were chosen at random at each time point over a period of 1 h (54.5 °C tests) or 48 h (24 and 35 °C tests). Virus was recovered by resuspension into culture medium and then quantified via a cell-based infectivity assay on Vero cells to determine the median tissue culture infectious dose (TCID_50_/mL). At each condition, a decay rate was derived from fitting a general linearlized model with a normal distribution and identity function to infectivity data from each trial over time. From these models, the time in hours required for 50% and 90% loss of starting infectivity was determined. Bars represent the means of results with individual replicates shown as points. Error bars indicate the standard deviation of each mean.

Our data indicate that high temperature negatively affects the stability of SARS-CoV-2 in saliva on surfaces. Previously, we showed that the times for 90% infectivity loss were approximately 52 and 21 h for virus at 24 °C (75 °F) and 35 °C (95 °F), respectively (Biryukov et al. 2020). In the present study, we show that a stainless steel surface present at an air temperature of 54.5 °C (130 °F) results in the inactivation of 90% of SARS-CoV-2 in approximately 0.6 h (36 min). In light of the COVID-19 pandemic, this information can be used to assess the potential for fomite transmission of SARS-CoV-2 and offer options to sanitize high-touch surfaces in situations where the temperature can be appropriately elevated.

One potential application of these results is thermal sanitization of unoccupied vehicles. Based on the limited information available in the Ford Motor Company press release, it is not possible to make a direct comparison between this study and the approach employed in Ford Police Interceptor Utility vehicles, yet our data support the use of active heating as a means to reduce the infectivity of SARS-CoV-2 on surfaces in vehicle interiors. Numerous studies have demonstrated the potential for passive heating of passenger cars to very high temperatures in as little as an hour when parked in sunlight with the windows up, although the final temperatures reached were dependent on the ambient temperature (McLaren et al. 2005; Grundstein et al. 2009; Vanos et al. 2018). Sunlight plays an important role in this heating process when short wave solar radiation transmitted through windows onto interior surfaces is partially reflected as long-wave thermal radiation, which effectively becomes trapped within the vehicle. In a study comparing vehicles parked in the sun versus the shade where the average ambient temperature was 38.8 °C (Tempe, AZ in summer), sun-exposed vehicles reached surfaces temperatures up to 68.9 °C in an hour, whereas shaded vehicles reached a maximal surface temperature of 47.8 °C. The effect of meteorological conditions on cabin temperature has been modeled (Horak et al. 2016), suggesting that such tools could potentially be leveraged in conjunction with virus decay data to estimate sanitization times and efficacy based on ambient conditions. Indeed, these data suggest that vehicle sanitization could be achieved in less than an hour under certain ambient conditions, which is a time frame that could feasibly be accomplished during routine errands (personal vehicles) or shift changes (for public transit vehicles).

Stainless steel coupons were utilized in this study and are likely relevant to surfaces commonly found on public transportation (seat backs, poles, hand rails, etc.). Our previous work showed that viral decay on three nonporous surfaces was similar (Biryukov et al. 2020), and so it is not unreasonable to anticipate the data presented here would be applicable to other nonporous surfaces as well. In contrast, there are conflicting results for viral decay on porous surfaces, with viral stability dependent on the surface type and generally tending to be less stable than nonporous surfaces (Chin et al. 2020; Kasloff et al. 2020; Riddell et al. 2020; van Doremalen et al. 2020). However, the efficiency of virus recovery from porous surfaces may be a contributing factor, but it does not appear that any study to date has controlled for physical virus recovery from such materials, potentially confounding these results. Additional studies would be needed to determine decay rates of virus on porous surfaces relevant to vehicle interiors under high temperature conditions.

## Conclusion

Overall, these data expand upon our previous findings that increasing temperature accelerates the decay rate of SARS-CoV-2 on surfaces and suggest that temperatures potentially encountered in vehicle interiors when parked outdoors during warmer months of the year may be sufficient to sanitize contaminated nonporous surfaces. When combined with normal cleaning/disinfection practices, this may further reduce the potential for fomite transmission of SARS-CoV-2. However, to fully assess the risk posed by SARS-CoV-2 surface contamination, additional information regarding the viable viral load present on surfaces, contact transfer efficiencies from surfaces, and the infectious dose of SARS-CoV-2 by relevant routes of exposure are needed.
